# Comparison of the predictive value of different non-insulin-based insulin resistance indices for acute kidney injury in patients with sepsis: a retrospective study

**DOI:** 10.3389/fendo.2025.1637119

**Published:** 2025-11-18

**Authors:** Shijie Wang, Ruowen Li, Li Zhang, Jiaqi Wang, Tingbin Xie, Xinying Wang

**Affiliations:** Clinical Nutrition Service Center, Department of General Surgery, Nanjing Jinling Hospital, Affiliated Hospital of Medical School, Nanjing University, Nanjing, China

**Keywords:** insulin resistance, surrogate markers, acute kidney injury, sepsis, critical care

## Abstract

**Objective:**

Insulin resistance (IR) is closely related to the development of acute kidney injury (AKI), but the preferred surrogate markers of IR have not been validated in patients with sepsis. This study aimed to evaluate the predictive value of triglyceride glucose index (TyG), metabolic score for IR (METS-IR) and triglyceride/high-density lipoprotein cholesterol (TG/HDL-C) for the development of AKI in patients with sepsis.

**Methods:**

Patients diagnosed with sepsis were retrospectively collected from the Medical Information Mart for Intensive Care IV (MIMIC-IV) database. The cohort was divided into based on the tertiles of the surrogate indices of IR and Kaplan-Meier curve was depicted the outcomes of each group. Correlations of these three surrogate markers of IR with sepsis-associated AKI and stage III AKI were evaluated through Cox regression models and restricted cubic spline (RCS).

**Results:**

Of the 997 septic patients enrolled in this study, 748 patients (75.03%) developed AKI and 286 (28.69%) eventually progressed to stage III AKI. Cox regression models and RCS showed that only METS-IR was significantly correlated with the development of AKI and stage III AKI in patients with sepsis in a non-linear positive manner. Subgroup analysis showed that the correlation between METS-IR and AKI was not remarkable in patients with heart failure (*P* = 0.925), chronic kidney disease (*P* = 0.284), and diabetes (*P* = 0.139).

**Conclusions:**

The current study compared the three accepted surrogate indices of IR and the results suggested that METS-IR may prove to be an ideal risk stratification tool for sepsis-associated AKI, showing a non-linear positive correlation.

## 1 Introduction

Sepsis is a life-threatening condition characterized by multi-organ damage due to dysregulation of the host’s immune response to infection (1). Globally, approximately 50 million new cases of sepsis are diagnosed each year, with a mortality rate of up to 20% (2). The kidney is one of the most common organs susceptible to sepsis-induced injuries, which in combination with acute kidney injury (AKI) substantially promote the mortality and long-term disability of the disease (3).

Systemic inflammation and microcirculatory disturbances in the organs were previously thought to be the key mechanisms leading to AKI, but metabolic disturbances during sepsis have attracted increasing attention in recent years (4). Sepsis is often accompanied with insulin resistance (IR) (5), which can inhibit the autophagic activity of podocytes, leading to kidney injury, and is positively correlated with kidney injury molecule-1 (6, 7). The high insulin-normal glucose clamp technique is considered as the gold standard for assessing IR, but the high cost and complexity of the assay procedure limit its large-scale application (8). Therefore, there is a growing interest in seeking easily accessible and low-cost surrogate indices of IR. Many previous studies have explored the relationship between triglyceride glucose index (TyG) and the development of AKI. Qin et al. found that TyG was independently associated with contrast-induced AKI (9), and Yang et al. reported that TyG had a certain predictive value for sepsis-related AKI (10). Metabolic score for IR (METS-IR), a novel indicator for evaluating insulin resistance, has been previously proven to be associated with estimated glomerular filtration rate (eGFR) and can serve as a monitoring index for chronic kidney disease (CKD) screening (11). Meanwhile, a high TG/HDL-C ratio has also been identified as an independent risk factor for CKD (12). Even in this context, no studies have so far explored the association between METS-IR, the TG/HDL-C ratio and AKI. It is necessary to find optimal surrogate indices of IR that could predict the occurrence of septic AKI and address which is more appropriate for clinical application. Compared with TyG and TG/HDL-C ratio, METS-IR not only includes biochemical indicators but also incorporates BMI, a crucial obesity index. BMI has been confirmed to be significantly associated with the development of AKI (13). Based on this, we hypothesize that METS-IR may exhibit the highest sensitivity for the prediction of sepsis-associated AKI.

The aim of the present study was to evaluate the predictive value of TyG and METS-IR and TG/HDL-C as three accepted surrogate markers of IR for predicting the risk of AKI in patients with sepsis, and determine which of them was more sensitive for identifying high-risk patients of AKI.

## 2 Methods

### 2.1 Study population

A retrospective observational design was conducted to obtain data from the Medical Information Mart for Intensive Care IV (MIMIC-IV) database, a large freely available database that includes high-quality medical records of more than 90,000 intensive care unit (ICU) admissions at Beth Israel Deaconess Medical Center (BIDMC) between 2008 and 2022. One author (WSJ) who had passed the tests of the Collaborative Institutional Training Initiative (CITI) gained permission to access the dataset, and was responsible for data extraction (Certification No. 56051808). The review committee of the Massachusetts Institute of Technology (MIT) and BIDMC approved the database for medical health-related research without requiring informed consent.

**Figure 1:**
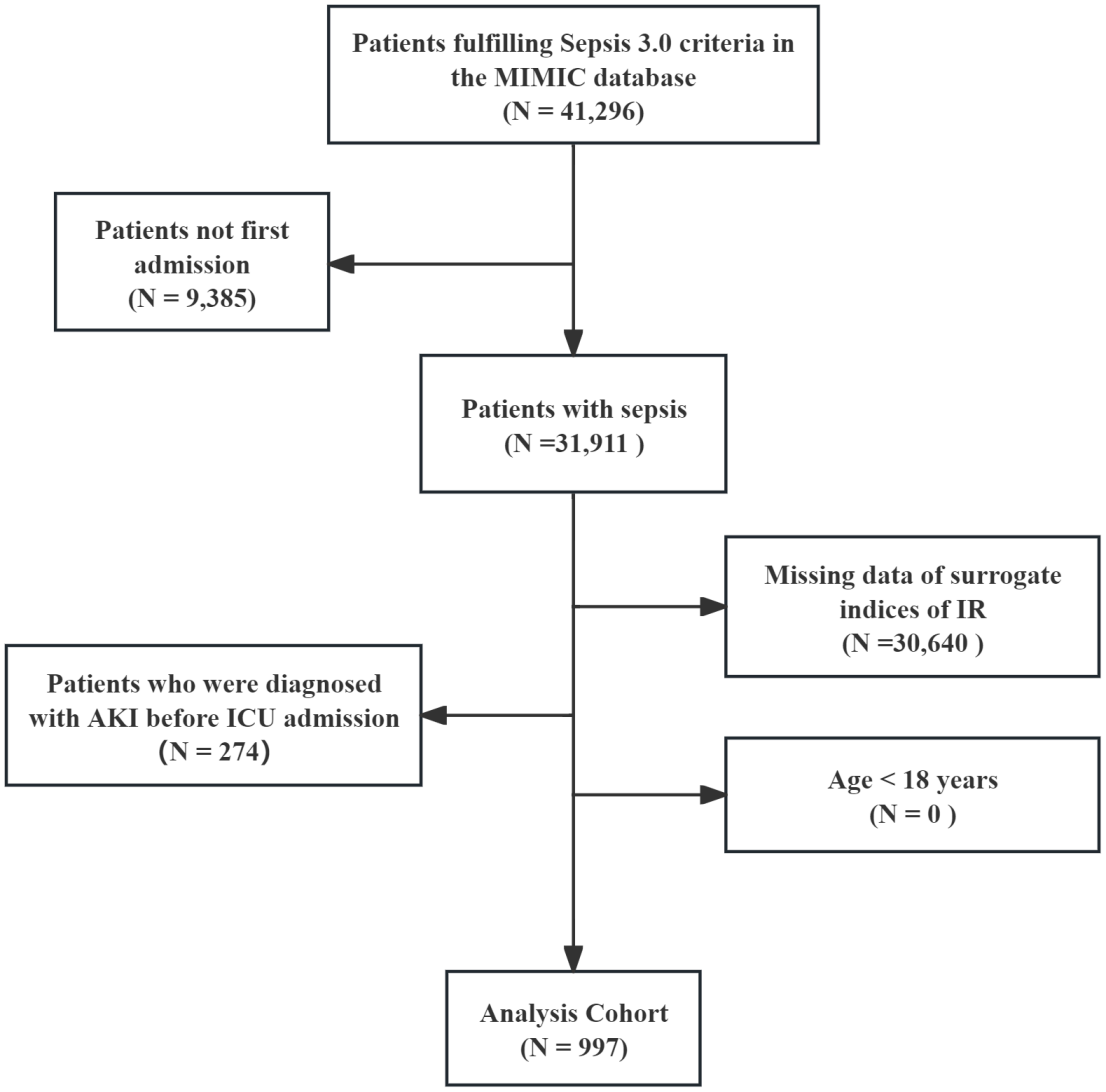
Flow diagram of patient selection.

All patients diagnosed with sepsis met the sepsis 3.0 diagnostic criteria (14), and the Kidney Disease: Improving Global Outcomes (KDIGO) guideline was used to confirm the presence of AKI (15). AKI was defined as follows: SCr increase ≥1.5-fold than baseline within 7 days; or SCr elevation ≥0.3 mg/dL within 48 hours; or urine output <0.5 mL/kg/h for ≥6 hours. Stage III AKI was defined as follows: SCr increase ≥>3-fold than baseline; or SCr ≥4.0 mg/dL; or receipt of RRT; or urine output <0.3 mL/kg/h for ≥24 hours or anuria for ≥12 hours. Patients with pre-existing mild AKI before ICU admission who progressed to Stage III AKI were excluded. The remaining exclusion criteria were as follows: 1) age less than 18 years; 2) only the first data were extracted if multiple ICU admissions for sepsis existed; 3) AKI was diagnosed before ICU admission; 4) missing data of surrogate indices of IR within 24 hours of ICU admission; and 5) missing AKI data within 48 hours. Finally, 997 patients with sepsis were enrolled and the cohort was divided into three groups based on the tertiles of the surrogate indices of IR (**Figure 1**).

### 2.2 Data collection

The basic characteristics of the patients were extracted from the MIMIC-IV database by PostgreSQL software (version 13.7.2) and Navicat Premium software (version 16) running a Structured Query Language (SQL). The primary data included: demographics, laboratory test results, comorbidities, treatment, medication, and severity of illness scores. Comorbidities were identified by the 9th and 10th revision editions of the International Classification of Diseases. All laboratory test results were extracted within the initial 24 h of ICU admission, and the serum creatinine (SCr) level and urine output were continuously monitored throughout the hospital stay. For the variables included in this study, those with missing values less than 20% were filled using multiple interpolation (MICE package), while those with missing values more than 20% were deleted. Procalcitonin, interleukin-6 (IL-6) and C-reactive protein (CRP) contained more than 20% missing value.

The surrogate indices of IR were formulated as follows:

TyG = ln [fasting TG (mg/dl) × fasting blood glucose (FBG) (mg/dL)/2] (10); METS-IR = ln[2 × TG (mg/dL) + FBG (mg/dL)] × BMI/ln HDL-C (mg/dL) (16); TG/HDL-C = TG (mg/dL)/HDL-C (mg/dL) (17).

### 2.3 Endpoints of interest

The primary endpoint of this study was the incidence of AKI, as defined by the KDIGO guidelines (18): an increase in SCr level by ≥1.5 times the baseline level within the previous 7 days; or an increase in SCr of ≥0.3 mg/dl within 48 h; or urine volume <0.5 ml/kg/h for 6 h or more. The baseline SCr was determined as the lowest value recorded within the 7 days prior to ICU admission; in the absence of such data, the initial SCr measurement upon ICU admission was utilized. The secondary endpoint was defined as the development of stage III AKI.

### 2.4 Statistical analysis

The proportional hazards assumption was verified using schoenfeld residual plots, and no violation was detected (**Supplementary Figure S1**).

In accordance with the Events Per Variable (EPV) principle, the sample size needs to be at least 10 times the number of variables. We included a total of 45 variables, corresponding to a required sample size of 450 cases. Meanwhile, in a study on TyG and sepsis-associated AKI, the incidence of AKI in TyG quartile groups was reported as 71.1%, 75.6%, 78.4%, and 88.8% (10). We hypothesize that the AKI incidence in the tertile groups of our study was 72%, 78%, and 85%. With a two-tailed type I error rate set at 5% and statistical power maintained at 90%, the minimum sample size required for the study was determined to be 750 patients (PASS version 15.0.5, NCSS, USA). With comprehensive consideration, at least 750 patients should be enrolled.

For continuous variables, Kolmogorov-Smirnov test was performed for normality, and Mann-Whitney U test or T-test was performed for group comparison. The categorical variables were assessed by Chi-square or Fisher exact tests. The occurrence of primary and secondary endpoints was depicted by the Kaplan–Meier curve and compared by the log-rank test. Knowing that feature selection is crucial for model construction, Boruta algorithm, a supervised categorical feature selection method, was used to determine all relevant features. The Fine-Gray model was constructed to analyze the competitive risk in order to evaluate the stability of the results. Knowing that the Cox proportional risk model can better reflect the effect of time to event as compared with logistic regression, it was used to assess the correlations between the surrogate indices of IR and the study endpoint. Three models were constructed: model 1: unadjusted; model 2: adjusted for age, sex and race; and model 3: adjusted for the specific results of the Boruta algorithm. To depict the dose-response relationship between surrogate indices of IR and risk of the study endpoint, restricted cubic spline (RCS) analysis was employed, with 4 knots specified based on variable distribution and set at the 5th, 35th, 65th, and 95th percentiles. Additionally, further stratified analyses were conducted according to age, gender, hypertension, heart failure (HF), coronary heart disease (CHD), chronic kidney disease (CKD), and diabetes. The interactions were assessed with likelihood ratio tests.

All statistical analyses were performed using R 4.1.3 (R Foundation for Statistical Computing, Vienna, Austria) and STATA 17.0 (STATA corporation, Texas, United States). *P* < 0.05 was considered statistically significant.

## 3 Results

### 3.1 Baseline characteristics

A total of 997 patients with sepsis were enrolled in the study, with a median age of 66.85 [57.88, 77.93] years, including 411 (41.22%) males. The median follow-up duration was 6 days. At the end of the follow-up period, AKI occurred in 748 patients (75.03%), and 286 (28.69%) eventually progressed to stage III AKI.

**Figure 2:**
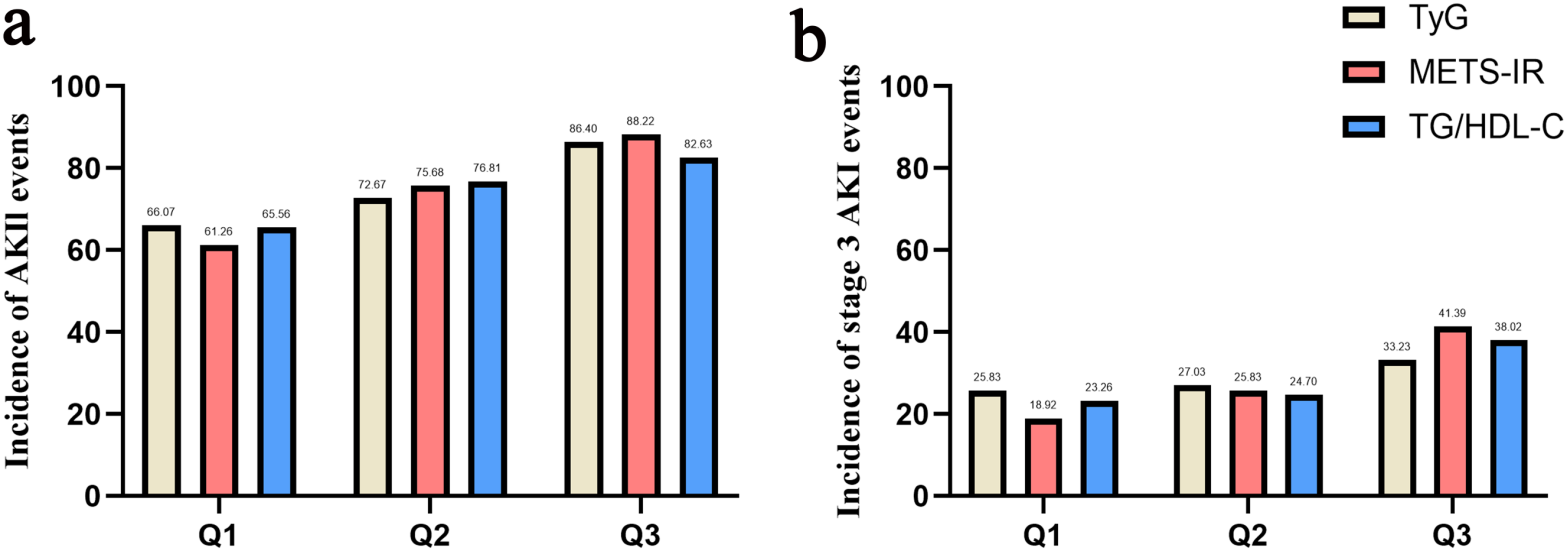
Bar graph of incidence of AKI **(a)** and stage III AKI **(b)** based on TyG index, METS-IR and TG/HDL-C.

The basic characteristics of the population stratified by TyG, METS-IR and TG/HDL-C were displayed in **Supplementary Table S1**. Numerous characteristics showed similar trends across the three indices, such that patients with higher indices tended to have higher BMI and SOFA score, higher prevalence of diabetes and septic shock, and higher usage rates of renal replacement therapy (RRT), meropenem and vancomycin. In addition, for laboratory markers, patients with higher indices tended to be associated with higher HDL, TG, SCr and glucose versus lower albumin and calcium. The incidence of AKI and stage III AKI in each group is shown as bar graphs in **Figure 2**. Clearly, the incidence of AKI, as well as stage III AKI, increased progressively from group Q1 to Q3 [TyG: AKI (66.07% vs 72.67% vs 86.40%, *P* < 0.001) and stage III AKI (25.83 vs 27.03 vs 33.23, *P* < 0.001); METS-IR: AKI (61.26% vs 75.68% vs 88.22%, *P* < 0.001) and stage III AKI (18.92% vs 25.83% vs 41.39%, *P* < 0.001); TG/HDL-C: AKI (65.56% vs 76.81% vs 82.63%, *P* < 0.001) and stage III AKI (23.26% vs 24.70% vs 38.02%, *P* < 0.001)].

Some inflammatory markers, such as CRP, were excluded owing to a high proportion of missing values. We therefore further compared the baseline characteristics between patients with and without CRP testing, and the results indicated no significant differences in baseline characteristics except for RRT and vancomycin (**Supplementary Figure S2**).

### 3.2 Feature selection

Boruta’s algorithm was used to perform feature screening of variables associated with AKI and stage III AKI (**Supplementary Figure S2**). In addition to the surrogate indices, the factors most strongly associated with AKI were, in order, body mass index (BMI), alanine aminotransferase (ALT), furosemide, aspartate aminotransferase (AST), age, glucose, SCr and insulin. The factors most strongly associated with stage III AKI were RRT, BMI, SCr, sequential organ failure assessment (SOFA) score, AST, blood urea nitrogen (BUN), bilirubin, hemoglobin, anion gap, hematocrit, septic shock, albumin, lymphocytes and ALT.

### 3.3 Correlations of the surrogate indices of IR with AKI

**Figure 3:**
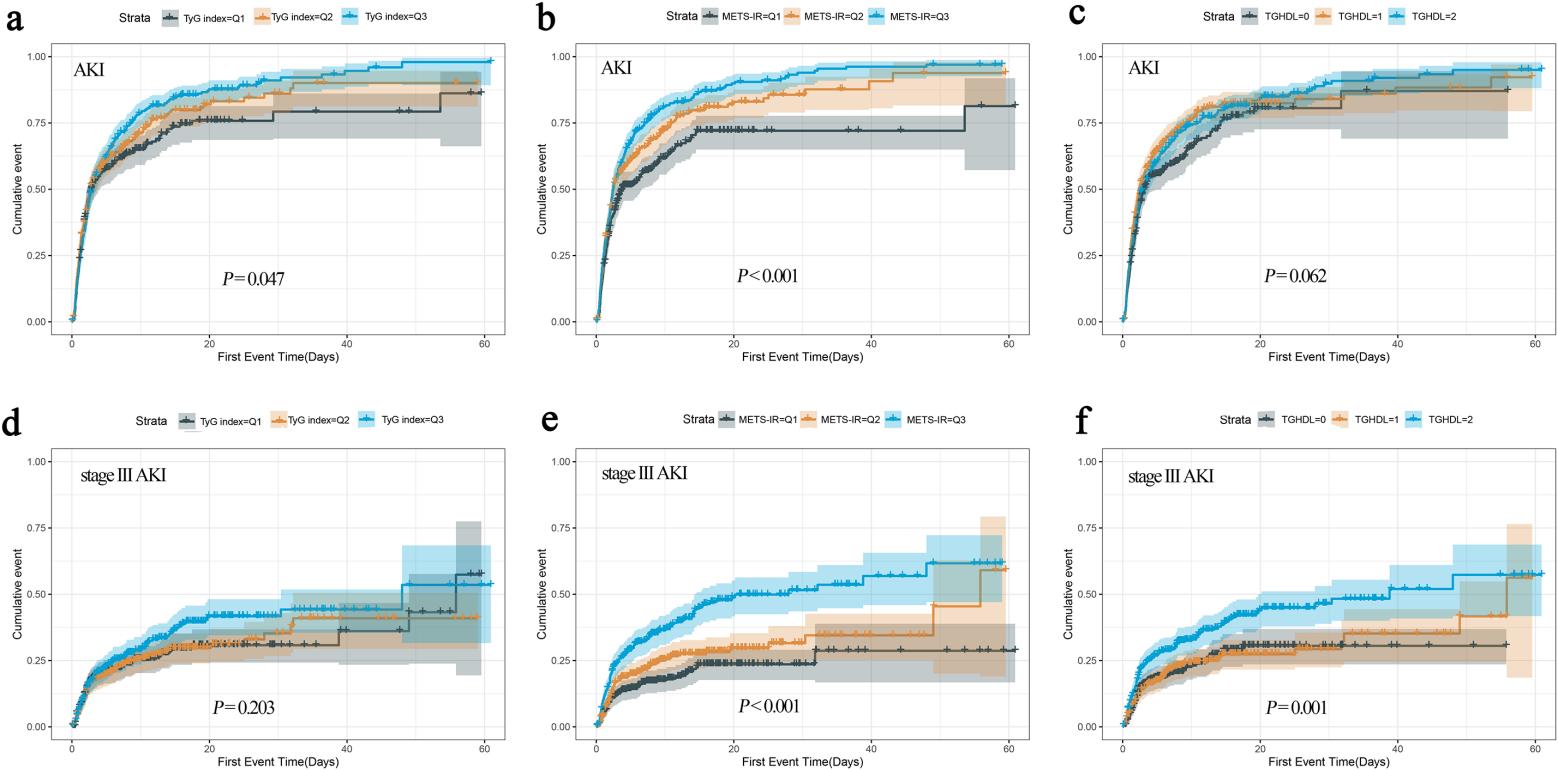
The cumulative event incidence curves for AKI based on TyG index **(a)**, METS-IR **(b)** and TG/HDL-C **(c)**. The cumulative event incidence curves for stage III AKI based on TyG index **(d)**, METS-IR **(e)** and TG/HDL-C **(f)**.

The occurrence of study endpoints was described by Kaplan-Meier method and compared by the log-rank test. (**Figure 3**). There was an overall significant difference in AKI incidence between the groups classified by TyG (log-rank χ²=6.106, *P* = 0.047) and METS-IR (log-rank χ²=24.709, *P* < 0.001), with patients in Q3 group having the highest incidence of AKI. However, when the groups were divided according to TG/HDL-C, no overall significant difference was observed (log-rank χ²=5.565, *P* = 0.062). Notably, for stage III AKI, an overall significant difference was observed between the groups divided by METS-IR (log-rank χ²=36.604, *P* < 0.001) and TG/HDL-C (log-rank χ²=15.102, *P* = 0.001), but not by TyG (log-rank χ²=3.188, *P* = 0.203).

Next, three Cox regression models were conducted to adjust for potential confounders: Model 1: unadjusted; Model 2: adjusted for age, sex and race; Model 3 (AKI): adjusted for age, sex, race, BMI, ALT, glucose, creatinine and furosemide; Model 3 (Stage III AKI): adjusted for age, sex, race, BMI, creatinine, AST, bilirubin, hemoglobin, anion gap, hematocrit, albumin, lymphocytes, RRT, septic shock and SOFA score. When METS-IR was used as a categorical variable in Models 3, the risk of AKI in the Q2 and Q3 groups was 31.3% and 52.4% higher than that in the Q1 group (*P* = 0.005 and *P* < 0.001, respectively), and the risk of stage III AKI was 40.2% and 85.5% higher (*P* = 0.043 and *P* < 0.001, respectively) (**Table 1**). Using TyG index as a categorical variable in Models 3, there was no overall significant difference between the groups for either AKI (*P* = 0.066) or stage III AKI (*P* = 0.855), although Q3 group showed a higher risk of AKI compared to Q1 group (*P* = 0.020) (**Table 1**). Meanwhile, there was also no significant difference between the groups for either AKI or stage III AKI for TG/HDL-C. Meanwhile, the results of the competitive risk analysis using the Fine-Gray model were similar to those of the cox regression analysis (**Supplementary Table S3**).

**Table 1:** The Cox regression analysis of surrogate indices of IR as categorical variables.

Categories	AKI		Stage III AKI	
	HR (95% CI)	*P*	HR (95% CI)	*P*
TyG index				
Model 1				
Q1	–	0.049	–	0.205
Q2	1.130 (0.941-1.356)	0.190	0.996 (0.741-1.339)	0.981
Q3	1.247 (1.046-1.486)	0.014	1.239 (0.935-1.644)	0.136
Model 2				
Q1	–	0.026	–	0.205
Q2	1.146 (0.954-1.377)	0.144	1.000 (0.744-1.345)	0.999
Q3	1.279 (1.070-1.528)	0.007	1.244 (0.936-1.654)	0.133
Model 3				
Q1	–	0.834	–	0.878
Q2	0.969 (0.713-1.317)	0.841	0.998 (0.738 ~ 1.349)	0.990
Q3	1.073 (0.772-1.491)	0.676	0.964 (0.716 ~ 1.297)	0.807
METS-IR				
Model 1				
Q1	–	<0.001	–	<0.001
Q2	1.320 (1.098-1.588)	0.003	1.372 (0.991-1.900)	0.056
Q3	1.566 (1.310-1.873)	<0.001	2.329 (1.728-3.139)	<0.001
Model 2				
Q1		<0.001	–	<0.001
Q2	1.330 (1.104-1.602)	0.003	1.346 (0.968-1.871)	0.077
Q3	1.609 (1.338-1.935)	<0.001	2.452 (1.803-3.336)	<0.001
Model 3				
Q1	–	<0.001	–	0.002
Q2	1.432 (1.026-1.999)	0.035	1.324 (0.952-1.842)	0.096
Q3	2.288 (1.664-3.146)	<0.001	1.838 (1.342-2.517)	<0.001
TG/HDL-C				
Model 1				
Q1	–	0.064	–	0.001
Q2	1.233 (1.029-1.478)	0.024	0.998 (0.730-1.362)	0.987
Q3	1.173 (0.981-1.403)	0.079	1.580 (1.190-2.100)	0.002
Model 2				
Q1	–	0.058	–	0.001
Q2	1.227 (1.024-1.472)	0.027	0.993 (0.726-1.358)	0.966
Q3	1.199 (0.999-1.439)	0.051	1.611 (1.205-2.153)	0.001
Model 3				
Q1	–	0.159	–	0.607
Q2	0.983 (0.717-1.346)	0.912	0.922 (0.672-1.265)	0.615
Q3	1.451 (1.082-1.945)	0.013	1.188 (0.872-1.617)	0.275

Model 1, unadjusted.

Model 2, adjusted for age, sex and race.

Model 3 (AKI), adjusted for age, sex, race, BMI, ALT, glucose, creatinine and furosemide.

Model 3 (Stage III AKI), adjusted for age, sex, race; BMI, creatinine; AST, bilirubin, hemoglobin, anion gap, hematocrit, albumin, lymphocytes; RRT, septic shock, and SOFA score.

When METS-IR was used as a continuous variable, the risk of AKI and stage III AKI increased with METS-IR increasing, whether in model 1, 2, or 3 (all *P* < 0.001). However, similar results were not shown for TyG and TG/HDL-C (all *P* > 0.05) (**Table 2**).

**Table 2:** The Cox regression analysis of surrogate indices of IR as continuous variables.

Categories	AKI		Stage III AKI	
	HR (95% CI)	*P*	HR (95% CI)	*P*
TyG index				
Model 1	1.029 (0.965-1.097)	0.386	1.058 (0.951-1.177)	0.303
Model 2	1.039 (0.975-1.109)	0.239	1.060 (0.951-1.181)	0.294
Model 3	1.034(0.890-1.200)	0.665	0.981 (0.847-1.135)	0.794
METS-IR				
Model 1	1.008 (1.005-1.011)	<0.001	1.014 (1.010-1.018)	<0.001
Model 2	1.009 (1.005-1.012)	<0.001	1.015 (1.011-1.019)	<0.001
Model 3	1.012 (1.008-1.016)	<0.001	1.009 (1.004-1.013)	<0.001
TG/HDL-C				
Model 1	1.000 (0.994-1.005)	0.886	1.008 (1.001-1.015)	0.036
Model 2	1.000 (0.994-1.006)	0.995	1.008 (1.001-1.016)	0.034
Model 3	1.002 (0.994-1.009)	0.648	0.996 (0.988-1.004)	0.276

Model 1, unadjusted.

Model 2, adjusted for age, sex and race.

Model 3 (AKI), adjusted for age, sex, race, BMI, ALT, glucose, creatinine and furosemide.

Model 3 (Stage III AKI), adjusted for age, sex, race, BMI, creatinine, AST, bilirubin, hemoglobin, anion gap, hematocrit, albumin, lymphocytes, RRT, septic shock and SOFA score.

**Figure 4:**
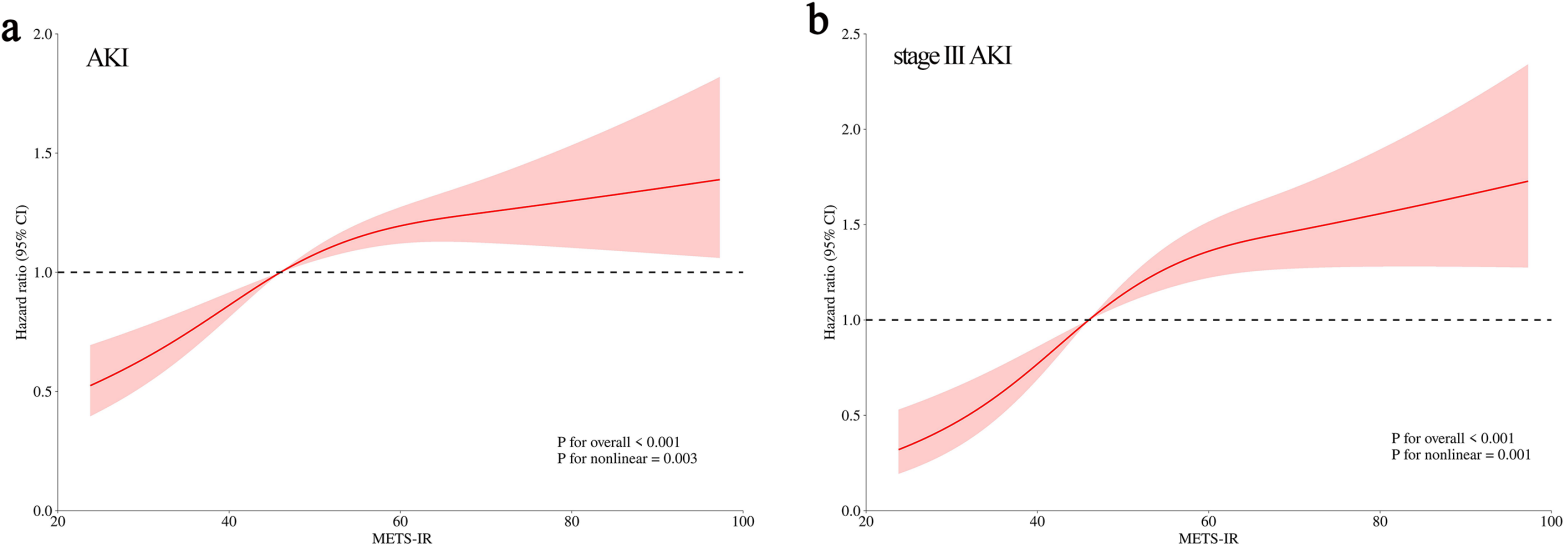
Restricted cubic spline analysis of METS-IR with AKI **(a)** and stage III AKI **(b)**.

Knowing that only METS-IR was independently correlated with the occurrence of AKI and stage III AKI as shown by Cox regression analysis, we further explored whether their relationship was linearly correlated by RCS curves. The results showed a gradual increase in AKI risk with METS-IR increasing in a non-linear positive manner (*P* for nonlinear = 0.003). A similar dose-dependent relationship was also observed between METS-IR and stage III AKI (*P* for nonlinear = 0.001) (**Figure 4**).

### 3.4 Stratified analysis

**Figure 5:**
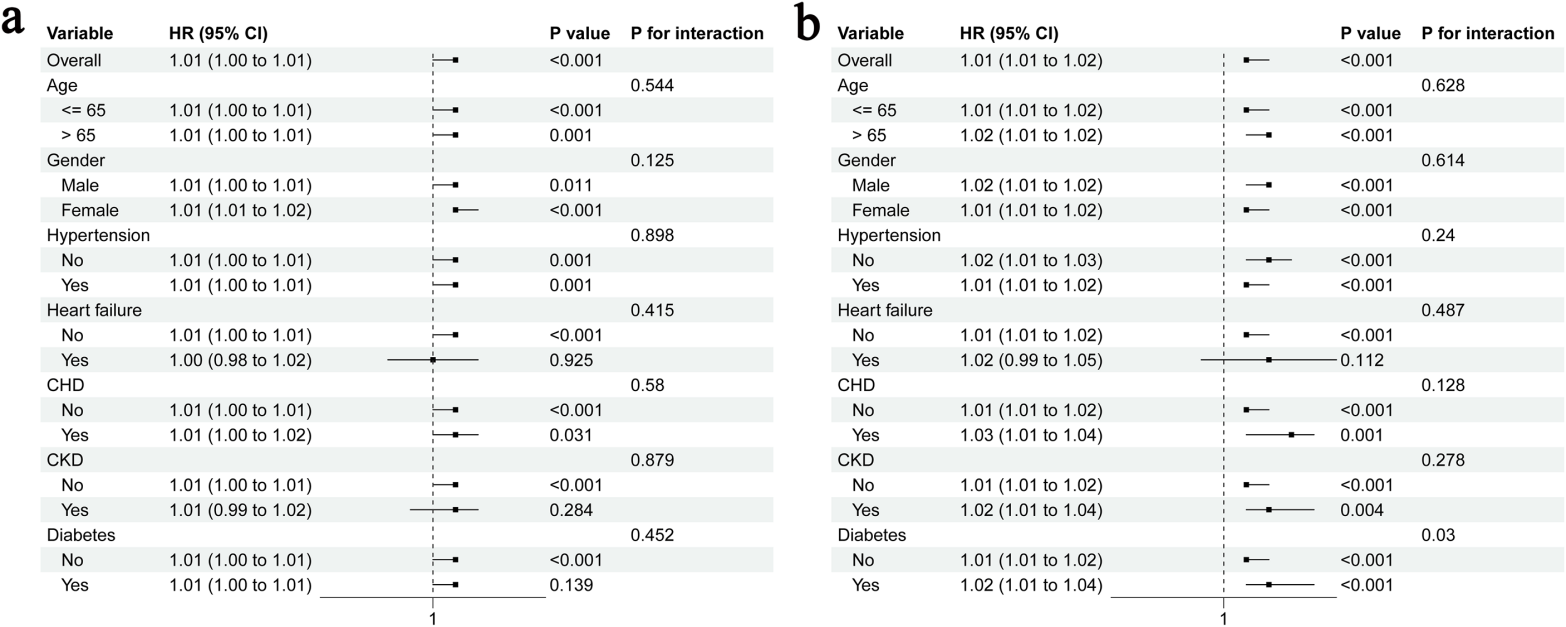
Stratified analyses for the association of METS-IR with AKI **(a)** and stage III AKI **(b)**; CHD, coronary heart disease; CKD, chronic kidney disease.

To examine whether the association between METS-IR and AKI persisted in specific populations, we performed stratified analyses according to age, gender, hypertension, HF, CHD, CKD and diabetes. The subgroups of age > 65 years or male sex included 553 and 411 patients, respectively. Meanwhile, the subgroups of hypertension, HF, CHD, CKD, and diabetes had 416, 67, 334, 155 and 346 patients, respectively. The results showed that METS-IR was strongly correlated with an increased risk of AKI in all populations except those with HF (*P* = 0.925), CKD (*P* = 0.284) and diabetes (*P* = 0.139), and there was no significant interaction between the subgroups (all *P* for interaction > 0.05) (**Figure 5A**). For stage III AKI, METS-IR was associated with an increased risk, except in patients with HF (*P* = 0.112) (**Figure 5B**).

## 4 Discussion

The current study compares the correlation between the three accepted surrogate indices (TyG, METS-IR and TG/HDL-C) of IR and the risk of AKI in patients with sepsis. Our findings demonstrated that METS-IR was significantly correlated with the occurrence of AKI and stage III AKI, providing a valid tool for assessing IR to optimize AKI risk stratification in critically ill patients with sepsis.

In the 997 included patients with sepsis, the AKI incidence was 75.03% and the stage III AKI incidence was 28.69%, which is consistent with the reported incidence of 14-87% (3). Such a high incidence of AKI not only leads to poorer survival and longer hospital stays but also long-term CKD (19). Therefore, early identification of patients predisposed to the development of AKI is extremely important, knowing that it could help focus attention and timely intervention. During sepsis, increased IR induced by inflammatory factors leads to hyperglycemia, which in turn induces increased ROS levels to damage renal tubular cells (20, 21). In addition, insulin-like growth factor-binding protein (IGFBP), an important factor involved in IR, has also been found to be directly involved in renal tubule injury (22). IR has been recognized as an important factor in the development or exacerbation of AKI (23).

The glucose clamp technique is the gold standard of IR, but it is expensive and not easily accessible. The emergence of surrogate indices of IR has solved this problem. Back in 2010, TyG was found to be highly consistent with the glucose clamp technique and could predict all-cause mortality in patients with type 2 diabetes (24, 25). In addition, a recent study explored the possible correlation between TyG and sepsis-related AKI (10), this study used logistic regression to assess the association between the TyG index and AKI. Although this method is applicable for cross-sectional or short-term outcome analyses, it neglects the variability in event occurrence time and censoring. In contrast, our study adopted Cox regression models - a method specifically designed for modeling time-dependent outcomes. This approach reduces bias caused by differences in follow-up duration and provides hazard ratios that better quantify the “time-varying risk” of outcomes. Two other indices, TG/HDL-C and METS-IR, have also been shown to predict individual insulin sensitivity (26, 27). Previous studies have shown their association with CKD, but the complex metabolic changes in sepsis may render these conclusions inapplicable to sepsis-associated AKI (11, 12). To date, their association with AKI remains uninvestigated, and no study has compared which of these three indices better predicts sepsis-associated AKI. In certain disease contexts, such as cardiovascular disease, METS-IR demonstrated a superior predictive ability for long-term patient survival than TyG and TG/HDL-C (27). Similarly, our study demonstrated that METS-IR exhibited significantly superior predictive capability compared to TyG and TG/HDL-C in relation to sepsis-associated AKI and stage III AKI. The risk of AKI and stage III AKI showed a non-linear gradual increase with the rising of METS-IR. Its strength in predicting septic AKI may derive from the fact that the METS-IR not only incorporates glucose, triglycerides, and HDL-C but also includes BMI. Notably, BMI has been identified to be associated with AKI. For example, a higher BMI is linked to an increased risk of AKI following major trauma (13), which may be attributed to adipose tissue releasing hormones under the stimulation of systemic inflammation, thereby exacerbating glomerular damage (28, 29). Therefore, the inclusion of BMI enables a more comprehensive assessment of the patient’s systemic condition.

To investigate which specific patients METS-IR is more applicable to, stratified analyses based on age, gender, hypertension, HF, CHD, CKD and diabetes were performed. we found that METS-IR was applicable for AKI across age, gender, and in patients with or without hypertension and CHD, but lost the predictive value in patients with comorbid HF, CKD, and diabetes. Possible explanations are that HF, CKD, and diabetes are important triggers for AKI (30–32). In these contexts, the role of sepsis-induced IR in the development of AKI may be partially masked. When the condition progresses to stage III AKI, its treatment and prognosis are quite different from those in patients with early AKI, which should arouse the clinician’s special attention (33). Prior research has reported that severe AKI constitutes 60% of AKI cases attributable to chronic HF, underscoring the profound influence of HF on renal impairment (34). This may also elucidate why the relationship between METS-IR and stage III AKI, as identified in our study, was masked within the context of HF. Nevertheless, for other patients, the routine detection of METS-IR at admission would facilitate the identification of patients at risk of AKI, thereby enabling the application of targeted preventive strategies.

The primary strength of this study lies in our validation of the predictive value of various surrogate indices of IR for sepsis-associated AKI. Our findings suggest that METS-IR emerges as the most promising option for clinical application, though certain limitations remain. Firstly, due to the retrospective design of the study, selection bias was inevitable. Secondly, certain critical clinical data, including the infection site, procalcitonin level and CRP were excluded from the study owing to insufficient information in the database. Given the association between inflammation markers and AKI, this may introduce some bias into the results. Thirdly, after stratification, the number of patients in subgroup analyses is relatively small, which may render the results of these subgroup analyses unreliable and thus require further validation with a larger sample size. Fourthly, the present study concentrated solely on evaluating the baseline values of the surrogate indices of IR, which may neglect dynamic changes occurring throughout the treatment period. Lastly, as the study was conducted at a single center, the findings may lack generalizability. Future prospective multicenter studies to address the above limitations and validate the findings are warranted.

## 5 Conclusions

The current study compared three accepted surrogate indices of IR and the results suggest that METS-IR may prove to be an ideal risk stratification tool for sepsis-associated AKI. Routine monitoring of METS-IR at admission could help clinicians identify patients at risk of AKI earlier and guide precise treatment.

## Data Availability

The original contributions presented in the study are included in the article/**Supplementary Material**. Further inquiries can be directed to the corresponding author.

## Supplementary material

Supplementary Figure 1 Visualization of Schoenfeld residuals.

Supplementary Figure 2 Feature selection process for AKI **(a)** and stage III AKI **(b)** risk based on Boruta’s algorithm.
