# Supplementary material for: Comparison of the predictive value of different non-insulin-based insulin resistance indices for acute kidney injury in patients with sepsis: a retrospective study
Source: Front Endocrinol (Lausanne). 2025 Nov 18;16:1637119. doi: 10.3389/fendo.2025.1637119 (PMC12668940; doi:10.3389/fendo.2025.1637119)
Supplement: Supplementary file 1 [file Table1.docx]

Table S1: The basic characteristics according to TyG index, METS-IR and TG/HDL-C.

|  | Overall | TyG index | | | | METS-IR | | | | TG/HDL-C | | | |
| --- | --- | --- | --- | --- | --- | --- | --- | --- | --- | --- | --- | --- | --- |
| Variables | Total (n = 997) | Q1 (n = 333) | Q2 (n = 333) | Q3 (n = 331) | *P* | Q1 (n = 333) | Q2 (n = 333) | Q3 (n = 331) | *P* | AIP (n = 331) | AIP (n = 332) | AIP (n = 334) | *P* |
| Age (years) | 66.85 (57.88, 77.93) | 69.16 (59.51,80.74) | 66.56 (58.25,78.62) | 65.67 (55.03,73.07) | <0.001 | 70.87 (60.94,82.13) | 68.45 (58.27,78.62) | 63.42 (55.12,71.50) | <0.001 | 69.23 (60.13,81.17) | 69.02 (60.31,79.54) | 63.56 (52.64,72.47) | <0.001 |
| Gender (male) | 411 (41.22) | 134 (40.24) | 144 (43.24) | 133 (40.18) | 0.656 | 160 (48.05) | 124 (37.24) | 127 (38.37) | 0.008 | 151 (45.62) | 128 (38.55) | 132 (39.52) | 0.134 |
| Height (cm) | 170.00 (163.00, 178.00) | 170.00 (163.00,178.00) | 170.00 (163.00,178.00) | 170.00 (163.00,178.00) | 0.768 | 168.00 (163.00,178.00) | 170.00 (163.00,178.00) | 173.00 (163.00,178.00) | 0.069 | 170.00 (160.00,178.00) | 170.00 (163.00,178.00) | 170.00 (163.00,178.00) | 0.382 |
| Weight (kg) | 80.99 (69.15, 97.95) | 77.50 (66.50,93.70) | 80.10 (69.60,95.30) | 85.60 (70.90,102.00) | <0.001 | 66.50 (57.60,74.60) | 80.80 (73.00,90.70) | 101.00 (88.67,116.00) | <0.001 | 77.10 (65.00,93.85) | 80.65 (70.45,99.03) | 84.75 (71.06,100.51) | <0.001 |
| BMI | 28.23 (24.53,32.82) | 27.14 (23.77,31.35) | 27.73 (24.54,32.86) | 29.95 (25.56, 33.95) | <0.001 | 23.44 (21.08,25.68) | 28.23 (26.05,30.46) | 34.60 (31.56,39.93) | <0.001 | 27.14 (23.39,31.35) | 28.11 (25.08,32.56) | 29.55 (25.39,33.74) | <0.001 |
| HDL (mg/dL) | 40.00 (30.00, 52.00) | 45.00 (33.00,60.00) | 40.00 (31.00,52.00) | 35.00 (25.50,44.00) | <0.001 | 52.00 (42.00,65.00) | 39.00 (31.00,47.00) | 32.00 (20.00,40.00) | <0.001 | 56.00 (46.00,67.00) | 40.00 (32.00,46.00) | 29.00 (19.00,37.00) | <0.001 |
| TG (mg/dL) | 108.00 (78.00, 158.00) | 68.00 (55.00,83.00) | 110.00 (94.00,131.00) | 192.00 (144.50,263.50) | <0.001 | 90.00 (67.00,121.00) | 106.00 (77.00,148.00) | 138.00 (99.00,223.00) | <0.001 | 70.00 (57.00,87.00) | 110.00 (89.00,133.00) | 192.00 (144.25,264.00) | <0.001 |
| Hematocrit (%) | 33.20 (27.60, 37.90) | 32.80 (26.50,37.80) | 33.80 (27.90,38.10) | 33.30 (28.60,37.80) | 0.573 | 33.80 (29.30,38.50) | 33.20 (27.20,37.40) | 32.60 (26.55,37.30) | 0.045 | 34.20 (29.45,38.50) | 33.55 (27.98,38.75) | 31.90 (25.92,36.70) | <0.001 |
| Hemoglobin (g/dL) | 10.90 (9.10, 12.50) | 10.80 (8.80,12.30) | 10.90 (9.00,12.60) | 11.00 (9.35,12.50) | 0.349 | 11.20 (9.50,12.70) | 10.80 (8.90,12.50) | 10.80 (8.90,12.40) | 0.046 | 11.20 (9.60,12.70) | 10.95 (9.20,12.62) | 10.30 (8.50,12.10) | <0.001 |
| Platelets (K/uL) | 173.00 (121.00, 233.00) | 167.00 (112.00,220.00) | 174.00 (126.00,237.00) | 177.00 (125.00,242.50) | 0.095 | 182.00 (136.00,244.00) | 169.00 (121.00,234.00) | 161.00 (104.00,224.00) | 0.003 | 180.00 (139.50,237.00) | 172.50 (124.00,231.50) | 157.00 (100.00,228.75) | 0.002 |
| WBC (K/uL) | 9.90 (7.40, 13.00) | 9.10 (6.60,12.10) | 9.90 (7.50,12.80) | 10.70 (8.15,14.20) | <0.001 | 9.60 (7.30,12.90) | 10.10 (7.20,13.00) | 10.00 (7.50,13.30) | 0.748 | 9.80 (7.50,12.60) | 9.70 (7.07,13.40) | 10.10 (7.53,13.28) | 0.732 |
| Albumin (g/dL) | 3.30 (2.80, 3.80) | 3.40 (2.90,3.80) | 3.40 (2.90,3.90) | 3.20 (2.70,3.70) | 0.008 | 3.50 (3.00,3.80) | 3.40 (2.80,3.80) | 3.10 (2.60,3.60) | <0.001 | 3.60 (3.20,3.90) | 3.30 (2.90,3.80) | 3.00 (2.50,3.50) | <0.001 |
| Anion gap (mEq/L) | 13.00 (11.00, 15.00) | 12.00 (10.00,15.00) | 13.00 (11.00,15.00) | 13.00 (11.00,15.00) | 0.007 | 13.00 (11.00,15.00) | 13.00 (11.00,15.00) | 13.00 (10.50,15.00) | 0.800 | 13.00 (11.00,15.00) | 13.00 (11.00,15.00) | 13.00 (10.25,15.00) | 0.720 |
| BUN (mg/dL) | 19.00 (13.00, 29.00) | 18.00 (13.00,29.00) | 19.00 (13.00,28.00) | 19.00 (13.00,29.50) | 0.336 | 17.00 (12.00,26.00) | 18.00 (13.00,27.00) | 21.00 (14.00,33.00) | <0.001 | 18.00 (12.00,26.00) | 19.00 (13.00,29.00) | 19.00 (13.00,31.75) | 0.117 |
| Creatinine (mg/dL) | 1.00 (0.70, 1.50) | 0.90 (0.70,1.40) | 1.00 (0.80,1.40) | 1.10 (0.80,1.55) | 0.034 | 0.90 (0.70,1.30) | 1.00 (0.80,1.40) | 1.10 (0.80,1.70) | <0.001 | 0.90 (0.70,1.30) | 1.00 (0.80,1.40) | 1.10 (0.80,1.60) | 0.023 |
| Glucose (mg/dL) | 118.00 (99.00, 143.00) | 104.00 (89.00,119.00) | 117.00 (103.00,139.00) | 140.00 (120.00,177.50) | <0.001 | 110.00 (94.00,134.00) | 120.00 (99.00,147.00) | 128.00 (106.50,151.00) | <0.001 | 116.00 (96.50,140.00) | 117.50 (100.00,143.25) | 122.00 (100.00,148.00) | 0.128 |
| Sodium (mEq/L) | 137.00 (134.00, 140.00) | 137.00 (134.00,139.00) | 138.00 (135.00,141.00) | 137.00 (134.00,140.00) | 0.171 | 138.00 (135.00,140.00) | 137.00 (134.00,140.00) | 137.00 (134.00,140.00) | 0.147 | 137.00 (134.00,140.00) | 138.00 (135.00,141.00) | 137.00 (134.00,140.00) | 0.028 |
| Potassium (mEq/L) | 3.80 (3.50, 4.20) | 3.80 (3.50,4.20) | 3.80 (3.50,4.10) | 3.80 (3.50,4.20) | 0.279 | 3.80 (3.50,4.10) | 3.80 (3.50,4.10) | 3.80 (3.50,4.20) | 0.588 | 3.80 (3.50,4.10) | 3.80 (3.50,4.12) | 3.80 (3.42,4.20) | 0.817 |
| Calcium (mg/dL) | 8.20 (7.70, 8.70) | 8.30 (7.70,8.80) | 8.30 (7.70,8.80) | 8.10 (7.70,8.60) | 0.029 | 8.30 (7.80,8.80) | 8.30 (7.70,8.70) | 8.10 (7.60,8.60) | 0.001 | 8.40 (7.90,8.95) | 8.30 (7.80,8.70) | 8.00 (7.50,8.50) | <0.001 |
| Chloride(mEq/L) | 102.00 (98.00, 105.00) | 102.00 (98.00,105.00) | 102.00 (99.00,105.00) | 102.00 (97.00,105.00) | 0.423 | 102.00 (99.00,105.00) | 102.00 (98.00,105.00) | 101.00 (97.00,105.00) | 0.075 | 102.00 (99.00,104.00) | 102.00 (99.00,106.00) | 102.00 (97.00,105.00) | 0.093 |
| Bicarbonate (mEq/L) | 21.00 (18.00, 23.00) | 21.00 (17.00,23.00) | 21.00 (18.00,24.00) | 20.00 (17.00,23.00) | 0.008 | 21.00 (18.00,23.00) | 20.00 (17.00,23.00) | 21.00 (17.50,23.00) | 0.410 | 21.00 (18.00,23.00) | 21.00 (18.00,23.00) | 20.00 (17.00,23.00) | 0.018 |
| Lymphocytes (K/µL) | 1.16 (0.74, 1.74) | 1.09 (0.70,1.67) | 1.16 (0.75,1.63) | 1.20 (0.76,1.92) | 0.167 | 1.11 (0.73,1.73) | 1.21 (0.74,1.81) | 1.16 (0.74,1.69) | 0.417 | 1.07 (0.73,1.62) | 1.18 (0.75,1.77) | 1.19 (0.76,1.90) | 0.207 |
| Monocytes (K/µL) | 0.69 (0.44, 1.07) | 0.67 (0.41,1.05) | 0.67 (0.42,1.05) | 0.73 (0.47,1.17) | 0.035 | 0.69 (0.40,1.06) | 0.68 (0.48,1.06) | 0.69 (0.44,1.10) | 0.869 | 0.68 (0.42,1.01) | 0.67 (0.44,1.06) | 0.73 (0.45,1.16) | 0.278 |
| Neutrophils (K/µL) | 9.26 (6.55, 14.26) | 8.92 (6.10,13.02) | 8.93 (6.53,13.70) | 10.08 (7.13,15.92) | 0.001 | 9.18 (6.50,14.26) | 9.03 (6.49,13.42) | 9.51 (7.04,15.02) | 0.210 | 9.42 (6.50,13.84) | 8.98 (6.51,13.96) | 9.46 (6.80,15.22) | 0.386 |
| INR | 1.20 (1.10, 1.40) | 1.20 (1.10,1.40) | 1.20 (1.10,1.30) | 1.20 (1.10,1.35) | 0.130 | 1.10 (1.00,1.30) | 1.20 (1.10,1.40) | 1.20 (1.10,1.40) | <0.001 | 1.10 (1.10,1.30) | 1.20 (1.10,1.40) | 1.20 (1.10,1.40) | <0.001 |
| PT (s) | 13.00 (11.90, 15.10) | 13.30 (11.90,15.60) | 12.90 (11.90,14.70) | 12.90 (11.90,14.75) | 0.354 | 12.40 (11.50,13.90) | 13.10 (12.10,15.30) | 13.60 (12.20,15.90) | <0.001 | 12.50 (11.65,14.30) | 13.10 (12.00,15.10) | 13.50 (12.10,15.90) | <0.001 |
| PTT (s) | 28.70 (25.40, 33.30) | 29.70 (26.00,34.60) | 28.40 (25.30,32.40) | 27.90 (25.25,32.30) | <0.001 | 28.40 (25.00,32.00) | 28.90 (25.70,35.30) | 28.90 (25.70,32.65) | 0.028 | 29.00 (25.50,33.85) | 27.80 (25.00,32.50) | 29.00 (26.10,34.18) | 0.038 |
| ALT (U/L) | 30.00 (17.00, 84.00) | 29.00 (16.00,74.00) | 29.00 (17.00,88.00) | 31.00 (18.00,91.00) | 0.444 | 25.00 (15.00,56.00) | 29.00 (17.00,83.00) | 37.00 (20.00,147.00) | <0.001 | 24.00 (16.00,53.50) | 29.00 (17.00,86.00) | 36.00 (20.00,146.50) | <0.001 |
| ALP (U/L) | 74.00 (58.00, 101.00) | 72.00 (57.00,101.00) | 71.00 (58.00,97.00) | 77.00 (60.00,103.50) | 0.025 | 75.00 (59.00,99.00) | 74.00 (59.00,98.00) | 72.00 (58.00,102.50) | 0.996 | 73.00 (58.00,97.00) | 72.00 (58.00,98.00) | 76.00 (59.00,105.00) | 0.110 |
| AST (U/L) | 44.00 (24.00, 138.00) | 41.00 (24.00,126.00) | 45.00 (24.00,139.00) | 48.00 (24.00,145.50) | 0.456 | 39.00 (23.00,100.00) | 44.00 (24.00,136.00) | 56.00 (26.00,197.00) | <0.001 | 34.00 (23.00,78.50) | 43.50 (24.00,133.25) | 67.00 (27.00,238.75) | <0.001 |
| Bilirubin (mg/dL) | 0.60 (0.40, 1.10) | 0.70 (0.40,1.20) | 0.60 (0.40,1.10) | 0.60 (0.40,1.15) | 0.674 | 0.50 (0.40,0.90) | 0.60 (0.40,1.20) | 0.70 (0.40,1.50) | <0.001 | 0.50 (0.40,0.80) | 0.60 (0.40,1.10) | 0.70 (0.40,1.80) | <0.001 |
| Hypertension n(%) | 416 (41.73) | 124 (37.24) | 152 (45.65) | 140 (42.30) | 0.086 | 144 (43.24) | 145 (43.54) | 127 (38.37) | 0.316 | 149 (45.02) | 148 (44.58) | 119 (35.63) | 0.021 |
| CHD n(%) | 334 (33.50) | 121 (36.34) | 104 (31.23) | 109 (32.93) | 0.364 | 108 (32.43) | 125 (37.54) | 101 (30.51) | 0.140 | 123 (37.16) | 113 (34.04) | 98 (29.34) | 0.099 |
| CKD n(%) | 155 (15.55) | 53 (15.92) | 53 (15.92) | 49 (14.80) | 0.901 | 52 (15.62) | 47 (14.11) | 56 (16.92) | 0.608 | 46 (13.90) | 64 (19.28) | 45 (13.47) | 0.071 |
| Heart failure n(%) | 67 (6.72) | 19 (5.71) | 27 (8.11) | 21 (6.34) | 0.439 | 21 (6.31) | 17 (5.11) | 29 (8.76) | 0.159 | 308 (93.05) | 308 (92.77) | 314 (94.01) | 0.798 |
| Diabetes n(%) | 346 (34.70) | 75 (22.52) | 114 (34.23) | 157 (47.43) | <0.001 | 87 (26.13) | 109 (32.73) | 150 (45.32) | <0.001 | 92 (27.79) | 117 (35.24) | 137 (41.02) | 0.002 |
| Septic shock n(%) | 133 (13.34) | 39 (11.71) | 33 (9.91) | 61 (18.43) | 0.003 | 30 (9.01) | 44 (13.21) | 59 (17.82) | 0.004 | 30 (9.06) | 42 (12.65) | 61 (18.26) | 0.002 |
| SOFA score | 3 (2,4) | 2 (2,4) | 3 (2,4) | 4 (2,5) | 0.016 | 3 (2,4) | 3 (2,4) | 3 (2,5) | <0.001 | 2 (2,4) | 3 (2,4) | 4 (2,5) | <0.001 |
| RRT n(%) | 150 (15.05) | 47 (14.11) | 39 (11.71) | 64 (19.34) | 0.019 | 29 (8.71) | 45 (13.51) | 76 (22.96) | <0.001 | 38 (11.48) | 40 (12.05) | 72 (21.56) | <0.001 |
| Furosemide n(%) | 676 (67.80) | 233 (69.97) | 220 (66.07) | 223 (67.37) | 0.548 | 196 (58.86) | 231 (69.37) | 249 (75.23) | <0.001 | 218 (65.86) | 230 (69.28) | 228 (68.26) | 0.627 |
| Spirolactone n(%) | 70 (7.02) | 28 (8.41) | 28 (8.41) | 14 (4.23) | 0.052 | 15 (4.50) | 28 (8.41) | 27 (8.16) | 0.088 | 14 (4.23) | 28 (8.43) | 28 (8.38) | 0.052 |
| Meropenem n(%) | 89 (8.93) | 23 (6.91) | 26 (7.81) | 40 (12.08) | 0.044 | 18 (5.41) | 31 (9.31) | 40 (12.08) | 0.010 | 18 (5.44) | 24 (7.23) | 47 (14.07) | <0.001 |
| Hydrochlorothiazide n(%) | 43 (4.31) | 11 (3.30) | 16 (4.80) | 16 (4.83) | 0.539 | 15 (4.50) | 13 (3.90) | 15 (4.53) | 0.903 | 15 (4.53) | 16 (4.82) | 12 (3.59) | 0.717 |
| Vancomycin n(%) | 758 (76.03) | 238 (71.47) | 254 (76.28) | 266 (80.36) | 0.027 | 228 (68.47) | 261 (78.38) | 269 (81.27) | <0.001 | 231 (69.79) | 256 (77.11) | 271 (81.14) | 0.002 |
| Cefepime n(%) | 420 (42.13) | 140 (42.04) | 128 (38.44) | 152 (45.92) | 0.149 | 136 (40.84) | 142 (42.64) | 142 (42.90) | 0.842 | 130 (39.27) | 139 (41.87) | 151 (45.21) | 0.299 |
| AKI n(%) | 748 (75.03) | 220 (66.07) | 242 (72.67) | 286 (86.40) | <0.001 | 204 (61.26) | 252 (75.68) | 292 (88.22) | <0.001 | 217 (65.56) | 255 (76.81) | 276 (82.63) | <0.001 |
| AKI III stage n(%) | 286 (28.69) | 86 (25.83) | 90 (27.03) | 110 (33.23) | <0.001 | 63 (18.92) | 86 (25.83) | 137 (41.39) | <0.001 | 77 (23.26) | 82 (24.70) | 127 (38.02) | <0.001 |

BMI: body mass index; HDL: high density lipoprotein; TG: triglyceride; WBC: white blood cell; BUN: blood urea nitrogen; INR: international normalized ratio; PT: prothrombin time; APTT: activated partial thromboplastin time; ALT: Alanine Aminotransferase; ALP: Alkaline Phosphatase; AST: Aspartate Aminotransferase; CHD, coronary heart disease; CKD, chronic kidney disease; RRT, renal replacement therapy; AKI, acute kidney injury; SOFA: sequential organ failure assessment; RRT, renal replacement therapy
