# Supplementary material for: Comparison of the predictive value of different non-insulin-based insulin resistance indices for acute kidney injury in patients with sepsis: a retrospective study
Source: Front Endocrinol (Lausanne). 2025 Nov 18;16:1637119. doi: 10.3389/fendo.2025.1637119 (PMC12668940; doi:10.3389/fendo.2025.1637119)
Supplement: Supplementary file 2 [file Table2.docx]

Table S2: Baseline characteristics of patients stratified by whether CRP test was performed.

| Variables | Total (n = 997) | no-CRP group (n = 367) | CRP group (n = 630) | *P* |
| --- | --- | --- | --- | --- |
| Age (years) | 66.85 (57.88, 77.93) | 66.85 (56.47, 77.30) | 66.80 (58.41, 78.00) | 0.512 |
| Gender (male) | 411 (41.22) | 151 (41.14) | 260 (41.27) | 0.969 |
| BMI | 28.23 (24.53, 32.82) | 28.53 (25.36, 32.94) | 27.99 (24.02, 32.76) | 0.116 |
| HDL (mg/dL) | 40.00 (30.00, 52.00) | 40.00 (29.00, 52.00) | 40.00 (31.00, 51.00) | 0.405 |
| TG (mg/dL) | 108.00 (78.00, 158.00) | 110.00 (78.50, 166.00) | 107.00 (77.00, 154.75) | 0.316 |
| Hematocrit (%) | 33.20 (27.60, 37.90) | 33.10 (28.25, 37.00) | 33.35 (27.15, 38.58) | 0.522 |
| Hemoglobin (g/dL) | 10.90 (9.10, 12.50) | 10.80 (9.20, 12.30) | 11.00 (9.00, 12.60) | 0.577 |
| Platelets (K/uL) | 173.00 (121.00, 233.00) | 169.00 (118.50, 232.00) | 175.50 (123.00, 236.25) | 0.451 |
| WBC (K/uL) | 9.90 (7.40, 13.00) | 9.70 (7.35, 13.20) | 10.05 (7.40, 12.90) | 0.830 |
| Albumin (g/dL) | 3.30 (2.80, 3.80) | 3.30 (2.90, 3.80) | 3.30 (2.80, 3.80) | 0.722 |
| Anion gap (mEq/L) | 13.00 (11.00, 15.00) | 13.00 (11.00, 16.00) | 13.00 (11.00, 15.00) | 0.365 |
| BUN (mg/dL) | 19.00 (13.00, 29.00) | 19.00 (13.00, 29.00) | 18.50 (13.00, 29.00) | 0.790 |
| Creatinine (mg/dL) | 1.00 (0.70, 1.50) | 1.00 (0.80, 1.50) | 1.00 (0.70, 1.40) | 0.247 |
| Glucose (mg/dL) | 118.00 (99.00, 143.00) | 120.00 (101.00, 146.50) | 117.00 (98.00, 142.00) | 0.260 |
| Sodium (mEq/L) | 137.00 (134.00, 140.00) | 137.00 (134.00, 140.00) | 137.00 (134.00, 140.00) | 0.640 |
| Potassium (mEq/L) | 3.80 (3.50, 4.20) | 3.80 (3.50, 4.10) | 3.80 (3.50, 4.20) | 0.766 |
| Calcium (mg/dL) | 8.20 (7.70, 8.70) | 8.20 (7.65, 8.65) | 8.20 (7.70, 8.80) | 0.057 |
| Chloride(mEq/L) | 102.00 (98.00, 105.00) | 102.00 (98.00, 105.00) | 102.00 (98.00, 105.00) | 0.565 |
| Bicarbonate (mEq/L) | 21.00 (18.00, 23.00) | 20.00 (17.50, 23.00) | 21.00 (18.00, 23.00) | 0.255 |
| Lymphocytes (K/µL) | 1.16 (0.74, 1.74) | 1.15 (0.73, 1.81) | 1.16 (0.74, 1.71) | 0.974 |
| Monocytes (K/µL) | 0.69 (0.44, 1.07) | 0.69 (0.46, 1.08) | 0.69 (0.42, 1.06) | 0.682 |
| Neutrophils (K/µL) | 9.26 (6.55, 14.26) | 9.21 (6.61, 14.84) | 9.30 (6.54, 13.90) | 0.734 |
| INR | 1.20 (1.10, 1.40) | 1.20 (1.10, 1.40) | 1.20 (1.10, 1.40) | 0.880 |
| PT (s) | 13.00 (11.90, 15.10) | 13.00 (12.00, 15.05) | 12.90 (11.83, 15.10) | 0.455 |
| PTT (s) | 28.70 (25.40, 33.30) | 28.40 (25.25, 32.95) | 28.80 (25.50, 33.40) | 0.497 |
| ALT (U/L) | 30.00 (17.00, 84.00) | 31.00 (18.00, 94.50) | 28.00 (17.00, 81.00) | 0.149 |
| AST (U/L) | 44.00 (24.00, 138.00) | 49.00 (25.00, 166.00) | 42.00 (24.00, 125.75) | 0.102 |
| Bilirubin (mg/dL) | 0.60 (0.40, 1.10) | 0.60 (0.40, 1.20) | 0.60 (0.40, 1.10) | 0.457 |
| Hypertension n(%) | 416 (41.73) | 161 (43.87) | 255 (40.48) | 0.295 |
| CHD n(%) | 334 (33.50) | 127 (34.60) | 207 (32.86) | 0.573 |
| CKD n(%) | 155 (15.55) | 50 (13.62) | 105 (16.67) | 0.201 |
| Heart failure n(%) | 67 (6.72) | 25 (6.81) | 42 (6.67) | 0.930 |
| Diabetes n(%) | 346 (34.70) | 126 (34.33) | 220 (34.92) | 0.851 |
| Septic shock n(%) | 120 (12.04) | 46 (12.53) | 74 (11.75) | 0.712 |
| SOFA score | 3.00 (2.00, 4.00) | 3.00 (2.00, 4.00) | 3.00 (2.00, 4.00) | 0.816 |
| RRT n(%) | 150 (15.05) | 84 (13.33) | 66 (17.98) | 0.048 |
| Furosemide n(%) | 676 (67.80) | 243 (66.21) | 433 (68.73) | 0.412 |
| Spirolactone n(%) | 70 (7.02) | 23 (6.27) | 47 (7.46) | 0.477 |
| Meropenem n(%) | 89 (8.93) | 29 (7.90) | 60 (9.52) | 0.386 |
| Hydrochlorothiazide n(%) | 43 (4.31) | 19 (5.18) | 24 (3.81) | 0.305 |
| Vancomycin n(%) | 758 (76.03) | 292 (79.56) | 466 (73.97) | 0.046 |
| Cefepime n(%) | 420 (42.13) | 147 (40.05) | 273 (43.33) | 0.312 |
| AKI n(%) | 748 (75.03) | 273 (74.39) | 475 (75.40) | 0.721 |
| AKI III stage n(%) | 286 (28.69) | 118 (32.15) | 168 (26.67) | 0.062 |

BMI: body mass index; HDL: high density lipoprotein; TG: triglyceride; WBC: white blood cell; BUN: blood urea nitrogen; INR: international normalized ratio; PT: prothrombin time; PTT: activated partial thromboplastin time; ALT: Alanine Aminotransferase; AST: Aspartate Aminotransferase; CHD, coronary heart disease; CKD, chronic kidney disease; RRT, renal replacement therapy; AKI, acute kidney injury; SOFA: sequential organ failure assessment; RRT, renal replacement therapy
