# Supplementary material for: Comparison of the predictive value of different non-insulin-based insulin resistance indices for acute kidney injury in patients with sepsis: a retrospective study
Source: Front Endocrinol (Lausanne). 2025 Nov 18;16:1637119. doi: 10.3389/fendo.2025.1637119 (PMC12668940; doi:10.3389/fendo.2025.1637119)
Supplement: Supplementary file 3 [file Table3.docx]

Table S3: The association between surrogate indices of IR and AKI and Stage III AKI based on Fine–Gray competing risk models.

| Categories | AKI |  | Stage III AKI |  |
| --- | --- | --- | --- | --- |
|  | HR (95% CI) | *P* | HR (95% CI) | *P* |
| TyG index |  |  |  |  |
| Model 1 |  |  |  |  |
| Q1 | - |  | - |  |
| Q2 | 1.130 (0.935-1.365) | 0.206 | 1.132 (0.935-1.371) | 0.204 |
| Q3 | 1.250 (1.047-1.481) | 0.014 | 1.293 (1.083-1.530) | 0.005 |
| Model 2 |  |  |  |  |
| Q1 | - |  | - |  |
| Q2 | 1.148 (0.954-1.393) | 0.144 | 1.149 (0.950-1.396) | 0.152 |
| Q3 | 1.278 (1.069-1.538) | 0.007 | 1.323 (1.107-1.584) | 0.002 |
| Model 3 |  |  |  |  |
| Q1 | - |  | - |  |
| Q2 | 1.071 (0.885-1.306) | 0.481 | 1.136 (0.937-1.377) | 0.194 |
| Q3 | 1.088 (0.892-1.332) | 0.405 | 1.280 (1.065-1.538) | 0.008 |
| METS-IR |  |  |  |  |
| Model 1 |  |  |  |  |
| Q1 | - |  | - |  |
| Q2 | 1.320 (1.096-1.590) | 0.003 | 1.342 (1.111-1.623) | 0.002 |
| Q3 | 1.572 (1.316-1.878) | < 0.001 | 1.645 (1.372-1.95) | < 0.001 |
| Model 2 |  |  |  |  |
| Q1 |  |  | - |  |
| Q2 | 1.330 (1.099-1.613) | 0.003 | 1.351 (1.115-1.641) | 0.002 |
| Q3 | 1.613 (1.344-1.947) | < 0.001 | 1.692 (1.413-2.034) | < 0.001 |
| Model 3 |  |  |  |  |
| Q1 | - |  | - |  |
| Q2 | 1.288 (1.061-1.566) | 0.011 | 1.336 (1.102-1.620) | 0.003 |
| Q3 | 1.507 (1.247-1.825) | < 0.001 | 1.638 (1.359-1.975) | < 0.001 |
| TG/HDL-C |  |  |  |  |
| Model 1 |  |  |  |  |
| Q1 | - |  | - |  |
| Q2 | 1.231 (1.027-1.481) | 0.025 | 1.271 (1.063-1.534) | 0.008 |
| Q3 | 1.175 (0.984-1.403) | 0.075 | 1.245 (1.042-1.470) | 0.016 |
| Model 2 |  |  |  |  |
| Q1 | - |  | - |  |
| Q2 | 1.228 (1.024-1.471) | 0.027 | 1.274 (1.060-1.534) | 0.010 |
| Q3 | 1.200 (1.001-1.442) | 0.048 | 1.023 (0.983-1.065) | 0.264 |
| Model 3 |  |  |  |  |
| Q1 | - |  | - |  |
| Q2 | 1.221 (1.017-1.476) | 0.032 | 1.247 (1.035-1.502) | 0.020 |
| Q3 | 1.172 (0.972-1.411) | 0.096 | 1.195 (0.984-1.452) | 0.072 |

Model 1: unadjusted;

Model 2: adjusted for age, sex and race;

Model 3 (AKI): adjusted for age, sex, race, BMI, ALT, glucose, creatinine and furosemide;

Model 3 (Stage III AKI): adjusted for age, sex, race, BMI, creatinine, AST, bilirubin, hemoglobin, anion gap, hematocrit, albumin, lymphocytes, RRT, septic shock, and SOFA score;
