# Supplementary figures and images for: Comparison of the predictive value of different non-insulin-based insulin resistance indices for acute kidney injury in patients with sepsis: a retrospective study
Source: Front Endocrinol (Lausanne). 2025 Nov 18;16:1637119. doi: 10.3389/fendo.2025.1637119 (PMC12668940; doi:10.3389/fendo.2025.1637119)

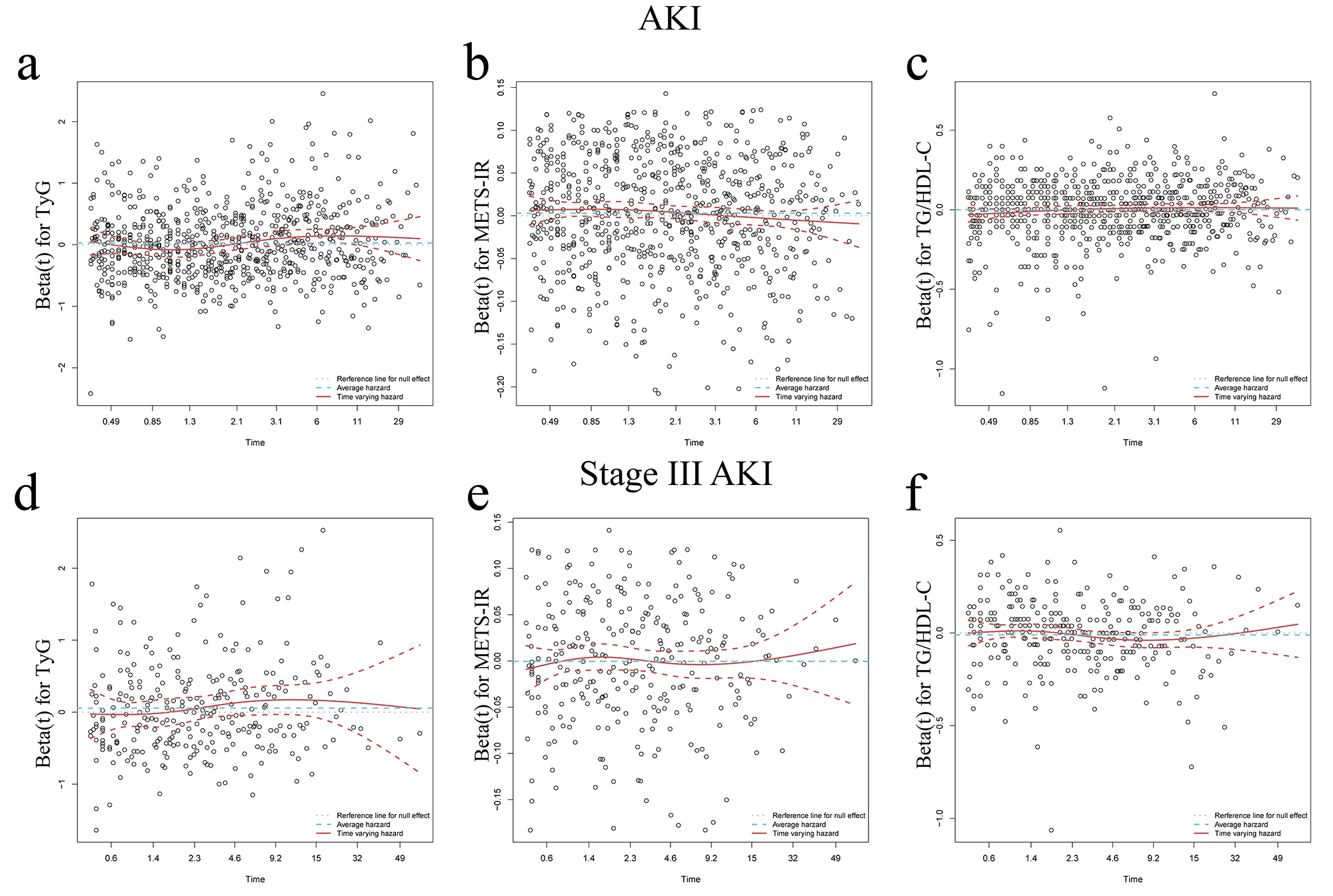

Supplement: Supplementary Figure 1 — Visualization of Schoenfeld residuals. [file Image1.tif]

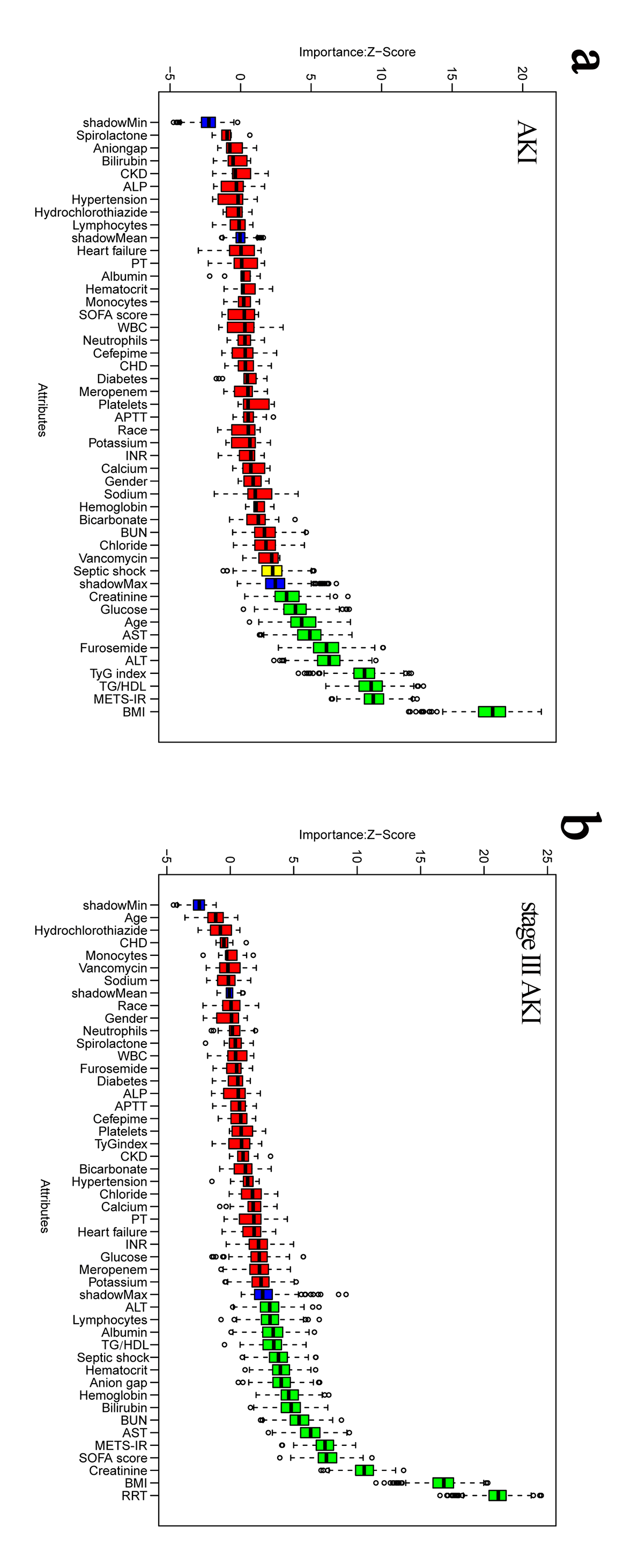

Supplement: Supplementary Figure 2 — Feature selection process for AKI (a) and stage III AKI (b) risk based on Boruta’s algorithm. [file Image2.tif]
